# T cell responses against tumor associated antigens and prognosis in colorectal cancer patients

**DOI:** 10.1186/1479-5876-3-3

**Published:** 2005-01-19

**Authors:** Dirk Nagorsen, Carmen Scheibenbogen, Anne Letsch, Christoph-Thomas Germer, Heinz-Johannes Buhr, Susanna Hegewisch-Becker, Licia Rivoltini, Eckhard Thiel, Ulrich Keilholz

**Affiliations:** 1 Medical Department III, Hematology, Oncology, and Transfusion Medicine, Charité University Medicine Berlin, Campus Benjamin Franklin, Berlin, Germany; 2 Department of Surgery, Charité University Medicine Berlin, Campus Benjamin Franklin, Berlin, Germany; 3 Department of Oncology and Hematology, University Clinic Eppendorf, Hamburg, Germany; 4 Istituto Nazionale Tumori, Milan, Italy

**Keywords:** T cell response, antigen, colorectal cancer, survival, prognosis

## Abstract

**Introduction:**

Spontaneous T cell responses against specific tumor-associated antigens (TAA) are frequently detected in peripheral blood of tumor patients of various histiotypes. However, little is known about whether these circulating, spontaneously occurring, TAA-reactive T cells influence the clinical course of disease.

**Methods:**

Fifty-four HLA-A2 positive colorectal cancer patients had been analyzed for the presence of T cell responses against epitopes derived from the TAA Ep-CAM, her-2/*neu*, and CEA either by ELISPOT assay or by intracellular cytokine staining. Then, Kaplan-Meier survival analysis was performed comparing T-cell-responders and T-cell-non-responders. For comparison, a group of T-cell-non-responders was compiled stringently matched to T-cell-responders based on clinical criteria and also analyzed for survival.

**Results:**

Sixteen out of 54 patients had a detectable T cell response against at least one of the three tested TAA. Two out of 21 patients (9.5%) with limited stage of disease (UICC I and II) and 14 out of 33 patients (42.4%) with advanced disease (UICC III and IV) were T cell response positive. Comparing all T-cell-responders (n = 16) and all T-cell-non-responders (n = 38), no survival difference was found. In an attempt to reduce the influence of confounding clinical factors, we then compared 16 responders and 16 non-responders in a matched group survival analysis; and again no survival difference was found (p = 0.7).

**Conclusion:**

In summary, we found no evidence that spontaneous peripheral T cell responses against HLA-A2-binding epitopes of CEA, her-2/neu and Ep-CAM are a strong prognostic factor for survival.

## Introduction

The importance of the immune system in containing tumor growth is supported by animal studies and various observations in humans [1,2]. These include increased prevalence of certain tumors following immunosuppression as well as the demonstration, that the presence of intralesional T cells is correlated with improved clinical outcome in various solid tumors [1,3-6]. In particular in CRC, the presence of CD8+ T cells within the tumor microenvironment was significantly associated with a better survival in several studies [3,7-9]. However, the antigen-specificity of these cells was not determined. T cell responses against specific tumor-associated antigens (TAA) are frequently detected in the peripheral blood of tumor patients [reviewed in [10]] of various histiotypes including colorectal cancer [11], melanoma [12,13], acute myeloid leukemia [14], breast cancer [15], neuroblastoma [16], and head and neck cancer [17]. Data from selected single patients suggest a favorable clinical course in patients with peripheral, spontaneous TAA-directed T cells [18,19]. However, this type of analysis does not allow firm conclusions. The only study comparing clinical outcome of patients with presence of antigen-specific immune responses including natural as well as vaccine-induced antibodies against melanoma antigen GM2 showed an improved survival in favor of immune responders [20]. TAA-directed T cell responses can reliably be induced using various vaccination approaches [reviewed in [21]]. Several recent reports have found a correlation between induction of a TAA-directed T cell response by vaccination and clinical response [22-25]. Preliminary data also suggest a possibly favorable clinical effect of vaccine-induced T cells in adjuvant vaccination [26-29]. Taken together, these data lead to the question, whether the presence of spontaneous TAA-specific T cells might be a positive prognostic factor. So far, however, no study has systematically compared survival data of patients with and without presence of a spontaneous TAA-directed T cell response.

In previous studies, we have demonstrated spontaneous T cell responses against the TAA CEA, Ep-CAM, or her-2/neu in peripheral blood of approximately 25% of colorectal cancer patients [11,30]. These cells were identified in functional T cell assays by antigen-induced IFNγ production. More detailed analyses in some samples revealed a CD3+ CD8+ IFNγ+ CD69+ CD45RA+ phenotype [11], indicative of an effector T cell subset that is able to directly mediate tumor cell lysis [19]. Spontaneous TAA-specific T cells with the potential of effector cells should, theoretically, be capable of destroying tumor cells and thereby lead to elimination of residual disease or prevent tumor progression. To investigate whether a peripheral, spontaneous T cell response has an effect on the clinical outcome of tumor patients, we analyzed survival data of CRC patients with a TAA-directed T cell response and compared these data with the clinical course of CRC patients without detectable T cell response.

## Patients, materials, and methods

### Patient selection and T cell assays

After institutional review board approval and informed consent, peripheral blood mononuclear cells from 132 patients with CRC in all stages of disease had been prospectively collected and frozen for T cell analysis. All analyses have been performed in compliance with the Helsinki Declaration. Fifty-four patients were tested positive for HLA-A2 and were subsequently analyzed for the presence of T cell responses against the HLA-A*0201 presented T cell epitopes Ep-CAM p263–271 [31], her-2/*neu *p654–662 [32,33], and CEA p571–579 [34] either by ELISPOT assay or by intracellular cytokine staining. HLA analysis, ELISPOT, and intracellular cytokine staining were performed as previously described [11,30]. Positive responses were defined as previously described [11,30].

### Survival analysis

First, we performed a Kaplan-Meier survival analysis comparing all T-cell-responders and all T-cell-non-responders. Additionally, a two-sided log rank test was used to test statistical significance. Then, in an attempt to reduce the influence of external factors, we compiled a patient group from non-responders matching them to responders according to the following criteria: UICC stage of disease, gender, presence of clinically detectable tumor at time of blood draw, duration of disease until blood draw, age at first diagnosis, and previous therapy. Survival in both groups was compared using Kaplan-Meier analysis and by a two-sided log rank test. A level of p < 0.05 was considered significant. SPSS (11.5) software was used.

## Results

### Patients, T cell response, survival of responders and non-responders

**Figure 1:**
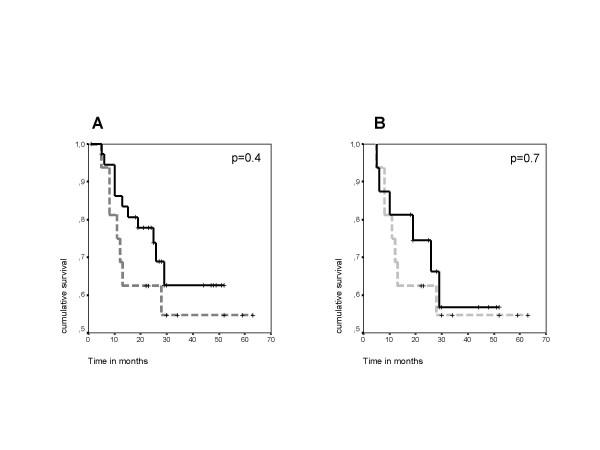
Kaplan-Meier survival analyses of colorectal cancer patients based on their T cell response state. Dashed lines refer to T-cell-responders, full lines refer to T-cell-non-responders, crosses mark censored cases. Time point 0 refers to the time of blood draw for T cell analysis. **A**. Two groups of CRC patients (total n = 54) were analyzed for survival after T cell analysis. One group had a spontaneous T cell response against the tumor associated antigens CEA, Ep-CAM, or her-2/neu (n = 16, dashed line). The other group had no T cell response against these antigens (n = 38, full line). No survival difference between the groups was found (log rank, p = 0.4). **B**. Two matched groups of CRC patients (total n = 32) were analyzed for survival after T cell analysis. One group had a spontaneous T cell response against tumor antigens CEA, Ep-CAM, or her-2/neu (n = 16, dashed line). The other group had no T cell response against these antigens and was selected by having similar clinical characteristics for stage, gender, presence of clinically detectable tumor at time of blood draw, duration of disease, age, and prior therapy (n = 16, full line). No survival difference was found (log rank, p = 0.7).

Fifty-four HLA-A2 positive CRC patients who had been analyzed for T cell responses were included in this study [11,30] and retrospectively analyzed for survival. The overall survival rate was 66.7% at a median of 27.5 months follow-up after blood draw for T cell analysis. In 16 out of 54 patients a total number of 26 T cell responses (between 10 and 1110 specific T cell per 10^6 ^PBMC) against one of the three TAA Ep-CAM, her-2/*neu*, and CEA had been detected. Comparing all responders (n = 16) and all non-responders (n = 38), no survival difference was found in a two-sided log rank test (p = 0.4; Fig. 1A). A very slight trend toward better survival was found in favor of non-responders, which is, however, far from being statistically significant. Since we have previously observed that T cell responses occur more frequently in patients with advanced stages of disease, we compared clinical data for all patients; and found that among non-responders only 50% (19 out of 38) had clinical stage III or IV disease while 87.5% (14 out of 16) responding patients had stage III or IV disease. Thus, the data on survival are possibly based on a strong stage-related bias. Therefore, we subsequently used an approach matching non-responders to responders.

### Survival in matched patient groups

Sixteen patients from the above group of 38 non-responders were matched with 16 responders to obtain two groups comparable for potentially confounding clinical factors, in particular stage of disease (see table 1). At the time of the survival analysis 13 of the total of 32 (40.6%) patients had died: seven T-cell-responders (n = 1 stage III, n = 6 stage IV) and six T-cell-non-responders (n = 1 stage III, n = 5 stage IV). All deaths were CRC-related. Median time to death among T-cell-responders was 11 months, among T-cell-non-responders 14.5 months. The calculated mean survival time after blood draw for patients without T cell response was 37.0 months (± 4.8 SEM) with a 95% confidence interval 27.5–46.5. Mean survival of T-cell-responders was 40.2 months (± 6.5 SEM) with 95% confidence interval of 27.5–52.9 (Fig. 1B). In a two-sided log rank test, survival did not show a statistically significant difference between responders and non-responders (p = 0.7). Of note, one to two years after blood draw, patients without T cell response had an up to 20% higher survival rate (approx. 80% vs. approx 60%). These results were, however, not significant. In a two-sided test with β = 0.2, a survival difference of 70% could have been considered significant at a level of α = 0.05 in a population of this size.

**Table 1:** Patient characteristics

**Peripheral spontaneous T cell response against TAA**		**Positive**	**Negative (matched)**	**Negative (total)**
Number of patients		16	16	38
Age at first diagnosis, mean ± SD		61.2 ± 12.1	59.9 ± 9.1	60.8 ± 11.1
Male/Female		11/5	11/5	19/19
Duration of disease until T cell analysis (mean)		31.8	30.2	27.4
Stage (UICC)	I	0	0	1 (2.6%)
	II	2 (12.5%)	2 (12.5%)	18 (47.4%)
	III	3 (18.8%)	3 (18.8%)	5 (13.2%)
	IV	11 (68.8%)	11 (68.8%)	14 (36.8%)
Previous therapy	CT	9 (56.3%)	12 (75%)	18 (47.4%)
	RT	3 (18.8%)	4 (25%)	7 (18.4%)
	Surgery	12 (75%)	14 (87.5%)	30 (78.9%)
Patients without evidence of tumor at time of blood draw		4 (25%, 2 II, 2 III)	4 (25%, 2 II, 2 III)	17 (45%, 13 II, 3 III, 1 IV)
Time from blood draw until death or last contact, mean ± SD		28.1 ± 19.2	27.4 ± 15.0	25.7 ± 14.0

Abbreviations: SD standard deviation, CT chemotherapy (5-FU + folic acid, in few cases plus Irinotecan), RT radiotherapy

## Discussion

In the present study, we analyzed the clinical course of colorectal cancer patients with or without T cells reactive against HLA-A2-binding epitopes of Ep-Cam, her-2/neu, and CEA. No survival difference between T-cell-responders and T-cell-non-responders was found. This result has to be interpreted cautiously due to the small number of HLA-A2+ patients responding to the above antigens. Obviously, these small numbers cause a high beta error. Thus, this study is only a first indication that the tested spontaneous T cell responses are not important prognostic factors for survival.

The second limitation of our study is that the known repertoire of TAA as potentially important T cell targets in CRC grows every year; and our T cell analysis included only a fraction of potential epitopes. Various other CRC-associated antigens, such as MUC1 or p53, and additional MHC class I antigenic epitopes of CEA and her2-neu have been described [summarized in [35]]. T cell responses against antigens in addition to the ones tested here could potentially play a role in immune surveillance of CRC.

Immune surveillance is understood as a complex process in which T cells and tumor cells influence each other in several ways ["immunoediting", [1]]. There are various potential factors related to tumor cells as well as T cells which may explain a lack of survival effect by TAA-specific T cells. The frequency of TAA-specific T cells detected in most patients was quite low in the range of 10 to 100 T cells per 10^6 ^PBMC. These numbers may be too low to control tumor growth especially in patients with a higher tumor burden. Furthermore, a general T cell dysfunction including anergic T cells and T cells with downregulated CD3-zeta chains has been described in CRC patients [36,37]. It is possible that the specific T cells detected in the present study are functionally unable to destroy tumor cells. This assumption, however, is not supported by our previous finding that TAA-specific T cells have an effector potential as analyzed in selected patients [11]. Since we have analyzed peripheral blood, we do not know if the circulating T cells have the potential to migrate to the tumor site or compartments where CRC cells frequently migrate to including lymph node, liver and bone marrow.

Furthermore, tumor cells may not be recognizable by TAA-specific T cells. It has been shown that CRC cell lines secrete immunosuppressive cytokines and that development of T cell responses is impeded due to low HLA expression and lack of intercellular adhesion molecule-1 (ICAM-1) and HLA-DR [38,39]. This is especially relevant considering the fact that TAA-specific T cell responses in peripheral blood are more frequently detectable in advanced stages of CRC [11,40], as well as other tumors [40,41]. These data led to the hypothesis that metastasizing of tumor cells to lymph nodes is a prerequisite for the development of circulating T cell responses [11,42]. Furthermore, the presence of TAA-directed T cell responses may have selected immune escape tumor variants. A broad variety of tumor escape mechanisms, such as antigen loss or loss of HLA expression, is described in various clinical conditions [43]. It is possible that we encounter similar mechanisms in the present study since malignant cells had grown *in vivo *during the presence of specific T cell responses. Finally, the role of suppressor and regulatory T cells in this specific context is unknown.

Taken together, no evidence was found that peripheral, spontaneous T cell responses against HLA-A*0201-binding epitopes of CEA, Ep-CAM, or her-2/neu influence survival of CRC patients. Since the low patient number limits the conclusion, further studies should investigate more patients, more detailed function and migratory pattern of spontaneous T cell responses as well as the genetic profile of the tumor; and consider a broader antigen and epitope repertoire. These studies could have implications for vaccination therapy as we learn more about why immune surveillance may fail to control tumors and if the presence of a natural T cell response may impact on the efficacy of a vaccine.

## Abbreviations

CEA carcinoembryonic antigen, CRC, colorectal carcinoma; ELISPOT, enzyme-linked immunospot; IFNγ, Interferon-γ; HLA, human leukocyte antigen; PBMC, peripheral blood mononuclear cells, TAA, tumor associated antigen.

## Competing interests

The author(s) declare that they have no competing interests.
